# Health Economics-Informed Social Return on Investment (SROI) Analysis of a Nature-Based Social Prescribing Craft and Horticulture Programme for Mental Health and Well-Being

**DOI:** 10.3390/ijerph22081184

**Published:** 2025-07-29

**Authors:** Holly Whiteley, Mary Lynch, Ned Hartfiel, Andrew Cuthbert, William Beharrell, Rhiannon Tudor Edwards

**Affiliations:** 1 Centre for Health Economics and Medicines Evaluation (CHEME), Bangor University, Bangor LL57 2PZ, UK; holly.whiteley@googlemail.com (H.W.); r.t.edwards@bangor.ac.uk (R.T.E.); 2 Faculty of Nursing and Midwifery, Royal College of Surgeons in Ireland, D02 YN77 Dublin, Ireland; maryalynch@rcsi.ie; 3 School of Medicine, Cardiff University, Cardiff CF14 4EP, UK; andrew@fathomtrust.com; 4 The Fathom Trust, Llanfellte Farm, Bwlch LD3 7JL, UK

**Keywords:** health economics, social return on investment (SROI), social cost–benefit, social value, nature based, social prescribing, green craft, horticulture, nature-based interventions (NBIs), mental health, well-being, prevention, forecast

## Abstract

Demand for mental health support has exerted unprecedented pressure on statutory services. Innovative solutions such as Green or Nature-Based Social Prescribing (NBSP) programmes may help address unmet need, improve access to personalised treatment, and support the sustainable delivery of primary services within a prevention model of population health. We piloted an innovative health economics-informed Social Return on Investment (SROI) analysis and forecast of a ‘Making Well’ therapeutic craft and horticulture programme for mental health between October 2021 and March 2022. Quantitative and qualitative outcome data were collected from participants with mild-to-moderate mental health conditions at baseline and nine-weeks follow-up using a range of validated measures, including the Short Warwick–Edinburgh Mental Well-being Scale, ICEpop CAPability measure for Adults (ICECAP-A), General Self-Efficacy Scale (GSES), and a bespoke Client Service Receipt Inventory (CSRI). The acceptability and feasibility of these measures were explored. Results indicate that the Making Well programme generated well-being-related social value in the range of British Pound Sterling (GBP) GBP 3.30 to GBP 4.70 for every GBP 1 invested. Our initial pilot forecast suggests that the programme has the potential to generate GBP 5.40 to GBP 7.70 for every GBP 1 invested as the programme is developed and delivered over a 12-month period. Despite the small sample size and lack of a control group, our results contribute to the evidence-base for the effectiveness and social return on investment of NBSP as a therapeutic intervention for improving health and well-being and provides an example of the use of health economic well-being outcome measures such as ICECAP-A and CSRIs in social value analysis. Combining SROI evaluation and forecast methodologies with validated quantitative outcome measures used in the field of health economics can provide valuable social cost–benefit evidence to decision-makers.

## 1. Introduction

### 1.1. Background

The COVID-19 pandemic and cost-of-living crisis have had a significant impact on population mental health and well-being, increasing the prevalence of anxiety and depression and putting additional strain on already overburdened health and social care systems [1,2,3,4,5]. 1.6 million people in the United Kingdom (UK) were on waiting lists for mental health treatment in 2022, with a further 8 million unable to access the list despite the service being considered beneficial for them [6]. Long wait times for services often result in further deteriorations in health and compound suffering [7,8]. Mental health conditions have been associated with poor quality of life, reduced workplace productivity, increased risk of physical illness, and higher mortality rates, all posing further challenge to health and social care systems and having wider economic impacts [9,10]. The economic cost of poor mental health in the UK is estimated to be at least GBP 118 billion [11].

The growing mental health crisis and its wide-ranging societal impact, coupled with limited resources and overstretched services, stresses the need for alternative approaches that provide accessible and cost-effective support, providing a positive social return on investment, to individuals living with long-term conditions like anxiety, depression, and stress [12]. Nature-based Social Prescribing (NBSP) of nature-based interventions (NBIs) to support mental health represents a potential solution and evidencing its impact, costs, and benefits for population health and well-being is the first step in enabling integration into policy and practice [13,14].

### 1.2. Nature-Based Social Prescribing (NBSP) to Support Mental Health

NBSP referrals connect individuals with a variety of non-clinical interventions and activities in natural settings designed to promote individual health and well-being, offering an alternative, holistic approach to supporting specific groups and/or people with health conditions [15,16]. NBSP of walking in nature, outdoor sports and gardening can reduce symptoms of stress, anxiety, and depression and promote good mental health and well-being [17,18,19,20].

Many NBI studies are small-scale and qualitative in nature, with large-scale randomised controlled trials (RCTs) of NBI clinical outcomes being rare due to cost and logistical difficulties. RCTs and quantitative-only approaches, however, may not be the most suitable methodology for NBI evaluation due to their complexity [21]. NBIs tend to be multi-faceted, require co-production with participants, and can deliver a range of outcomes [14,22]. Evaluating these complex interventions requires a broad approach that considers all impacts, the mechanisms through which benefits are realised, and that generates appropriate evidence to support effective and cost-effective real-world implementation yielding a positive social return on investment [23]. In general, NBSP provides broad social and environmental benefits; however, its implementation encounters challenges. To be effective, NBSPs require context-specific, standardised, and adaptable protocols for both implementation and evaluation 

Here an innovative health economics-informed mixed-method Social Return on Investment (SROI) evaluation and forecast of a nine-week ‘Making Well: Health & Healing through green crafts’ therapeutic NBSP programme (hereafter MW programme) explored the social cost–benefit and social value generated for relevant stakeholders. The acceptability and feasibility of using a range of quantitative health economics outcome measures to inform SROI estimates were investigated.

### 1.3. The Fathom Trust MW Programme

The MW programme is delivered by The Fathom Trust (hereafter Fathom), a charitable organisation that works closely with a network of public and private partners, including local landowners, Brecon Beacons National Park Authority, the National Health Service (NHS), and local authority, to provide safe environments in which people can learn and take part in practical nature-based tasks that are mentally relaxing and create opportunities for developing new skills, relationships and perspectives to promoting good health and well-being. The MW programme consists of a one-day taster session followed by an 8-week structured programme delivered over nine consecutive weeks (i.e., nine sessions in total). The programme brings together green crafts, therapeutic horticulture, and mindfulness in natural settings to provide therapeutic, nature-based support for individuals with long-term mild-to-moderate mental health conditions.

Our pilot health economics-informed SROI evaluation and forecast of the MW programme took place over six-months between October 2021 and March 2022. The pilot collected data from participants that attended two nine-week MW programmes delivered one after the other between October 2021 and March 2022.

The study was sponsored by the Accelerate programme, a healthcare innovation programme led by the Life Science Hub Wales in partnership with Cardiff University, Swansea University and University of Wales Trinity Saint David, and part-funded by the European Regional Development Fund (ERDF). Ethical approval was gained from Bangor University Healthcare and Medical Sciences Academic Ethics Committee in October 2021 and the SROI evaluation and forecast was undertaken by researchers at the Social Value Hub at the Centre for Health Economics and Medicines Evaluation (CHEME), Bangor University, Wales UK.

## 2. Materials and Methods

### 2.1. Social Return on Investment (SROI) Methodology

SROI is a pragmatic, mixed-method form of Social CBA that explores the social costs and outcomes of activities from the perspective of the people and organisations that experience them [24]. Both National Institute for Health and Care Excellence (NICE) and HM Treasury endorse the use of Social CBA for the evaluation of public health and well-being interventions [25]. The Medical Research Council (MRC) framework for evaluating complex interventions highlights six core elements: consider context, develop and refine programme theory, engage stakeholders, identify key uncertainties, refine the intervention, and include economic considerations [26]. These core elements align with the SROI methodology.

SROI considers all relevant costs and intended and unintended outcomes of an intervention and assigns them a market or financial proxy value using appropriate valuation techniques [27]. The social value of identified outcomes is compared with the intervention costs to estimate an SROI ratio that demonstrates value for money in terms of the amount of social value created per GBP 1 invested.

In this health economics-informed SROI evaluation and forecast we apply the six key stages underpinning a robust SROI evaluation: (i) identifying key stakeholders; (ii) mapping inputs, outputs and outcomes; (iii) evidencing and valuing outcomes; (iv) establishing impact; (v) calculating the SROI; and (vi) reporting, using and embedding the results [24]. Alongside these stages we also applied the HM Treasury Green Book guidance on well-being valuation [27] to evaluating the impact and social cost–benefit of the MW programme. The MW programme inputs (i.e., costs), outputs (i.e., intervention activities), and relevant stakeholder outcomes (i.e., benefits) were identified and valued, and an SROI ratio comparing the cost per participant with the social value generated per participant was estimated. The social cost–benefit evidence was then incorporated into an SROI forecast of the social value expected to be generated by the MW programme in subsequent years.

### 2.2. Identifying Stakeholders

Identifying stakeholders sets the perspective of an SROI analysis. In heath economics, perspective determines the breath of outcomes and impacts considered. In this pilot SROI evaluation and forecast, the MW programme participants were considered the primary stakeholders because they directly engaged with the programme and were expected to experience the most outcomes. NHS Wales were considered secondary stakeholders as they may also benefit from the programme if participants reduced their health service use due to involvement in the programme.

MW programme participants included people over the age of 18 years who had personal experience of a chronic mild-to-moderate mental health condition and an interest in developing skills in horticulture, craft-making, and/or self-care. No previous gardening or crafting experience was required and all activities were designed to be fully accessible. Participants were referred to the programme by General Practitioners (GPs), community mental health teams and third-sector organisations (e.g., MIND).

Participant eligibility inclusion criteria included the following key requirements: participants were aged 18 years and above; were referred to the programme because they were experiencing a long-term mild-to-moderate mental health condition; were able to speak English or Welsh; and had the mental capacity to provide informed consent, reflect on their own well-being, and identify the impacts of participating in the MW programme.

There was space for 10 participants on each nine-week MW programme. 20 participants in total enrolled onto the two MW programmes that were delivered during this pilot study. 19 out of 20 participants opted to complete a baseline questionnaire at the start of the study. Further information on completion rates, participant baseline demographic characteristics and self-reported outcomes can be found in Section 3.2: Stakeholder outcomes.

### 2.3. Mapping Inputs, Outputs and Outcomes

**Figure 1:**
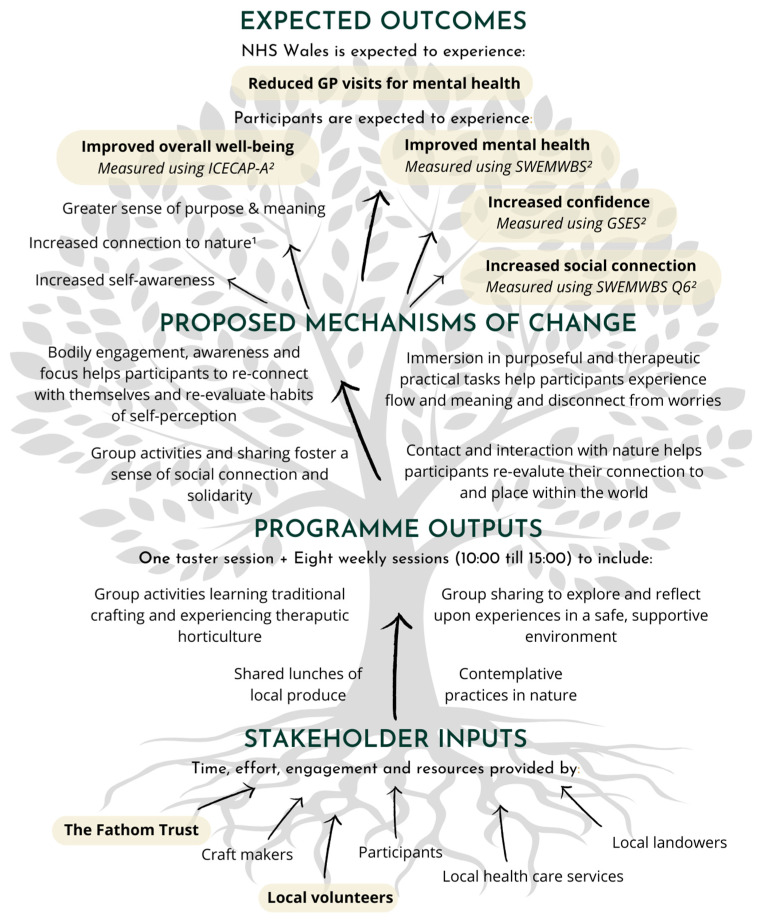
**Theory of change model for the Making Well programme.** Emboldened and highlighted inputs and outcomes indicate the inputs and outcomes included and valued in the SROI evaluation. ^1^ Connection to nature was measured in SROI evaluation but not valued due to a lack of available valuation techniques and financial proxies for this outcome. ^2^ Measures used to quantify changes in expected outcome included the ICEpop CAPability measure for Adults (ICECAP-A) instrument, Short Warwick–Edinburgh Mental Well-being Scale (SWEMWBS) and General Self-Efficacy Scale (GSES).

Theory of change models explore and illustrate the underlying assumptions and linkages between inputs, outputs and expected outcomes of a programme [28]. A theory of change model was developed to map and explore the MW programme inputs, outputs and expected outcomes for programme participants and NHS Wales (Figure 1). The theory of change model also outlines the proposed mechanisms of change that link programme outputs to expected outcomes. The theory of change model was co-produced with Fathom representatives, previous Fathom beneficiaries and the instructors that led the MW programme sessions and informed the selection of outcome measures included in the MW programme participant questionnaires. The inputs, outputs and expected outcomes of the MW programme are presented in further detail in Section 2.3.1, Section 2.3.2 and Section 2.3.3. The outcomes measures used to quantify changes in expected outcomes are discussed in Section 2.4 Evidencing and valuing outcomes.

The proposed mechanisms of change presented in Figure 1 are based on Fathom’s programme aims and objectives plus supporting literature that explores participant experiences of nature-based ‘green care’ [29], its theoretical underpinnings [30], and the benefits of nature-connectedness [31]. Group craft and therapeutic horticulture activities and sharing lunch, for example, were expected to help participants experience social connection and solidarity [29]. Contact and proactive interaction with nature was expected to help participants re-evaluate their connection to, and place within, the natural world and experience the documented positive impact of nature on overall well-being and mental health [30,31]. Immersion in purposeful and therapeutic practical work was expected to help participants experience a sense of flow and meaning as well as an opportunity to disconnect from concerns and worries, helping to support overall well-being and mental health [29,30]. Finally, learning new skills and overcoming challenges was expected to help participants build self-confidence and self-worth [29]. Exploring the causal links between the above-proposed mechanisms of change and stakeholder outcomes was beyond the scope of SROI methodology used in this study.

#### 2.3.1. The MW Programme Inputs

The MW programme inputs and associated costs were identified through consultation with Fathom. These inputs included organisational overheads (e.g., governance, management, administration, site hire costs), nature-based session costs (e.g., green craft, therapeutic horticulture, outdoor heating costs), catering, support staff, and volunteer contributions.

**Governance:** Fathom’s Board of Trustees provide oversight, governance, and strategic direction for the charity. Trustee Board meetings are held online four times per year for approximately one hour per meeting. Volunteer Trustees offer their time for free and are altruistic in support of the charity’s development. To ensure transparency in Trustee volunteer opportunity costs, hourly rates (based on 235 working days per year and 7.5 working hours per day) were calculated for Trustees using job titles and estimated average salaries in the UK from payscale.com [32,33]. Where salary estimates were not available from payscale.com, estimates obtained from the NHS were used [34].

**Management and administration:** For the six-month MW programme pilot project, the management and administration of the MW programme was undertaken by Fathom’s Director. An average Executive Director of Non-profit organisations salary per annum sourced from [32], and an assumption of 253 working days per year and 7.5 h per day, was used to estimate an hourly rate GBP 23.40 per hour (rounded to nearest ten pence) for this input.

**Site rent:** For the six-month MW programme pilot project the chosen location was Llanfellte Farm in the Brecon Beacons, Wales at a cost of GBP 100 per day including use of utilities.

**Green crafts and therapeutic horticulture:** Green craft and therapeutic horticulture sessions are central to the MW programme. The MW programme taster day included three craft sessions. The subsequent eight-week programme (eight 5 h days) comprised two craft sessions per day at an estimated cost of GBP 250 per session. This included freelance artisan crafter fees, tools, and materials.

**Outdoor heating:** The MW programme took place between October 2021 and March 2022, with most activities taking place outside. Outdoor heating for participants and staff was provided by fire bowls. Fire bowls and firewood were provided by Fathom at a cost of GBP 150 per fire bowl and GBP 20 worth of firewood per programme.

**Catering:** Lunch and light refreshments were provided for MW programme participants, staff, and volunteers. The Fathom Trust provided an estimated cost of GBP 7.50 per person per day.

**Support staff:** A Social Work Assistant employed by MIND accompanied participants on the MW programme. The role of the Social Work Assistant was to provide independent, one-to-one support to participants where required. An average Community Support Worker salary per annum sourced from [payscale.com], and an assumption of 253 working days per year and 7.5 h per day, was used to estimate an hourly rate of GBP 12.20 per hour for this input.

**Volunteer time:** Volunteers supported MW programme delivery by preparing and tidying workspaces and organising and serving food and refreshments. Ten hours of volunteer time per week were required to run the nine-week MW programme. All volunteers involved were retired and their volunteer opportunity cost was estimated using the National Living Wage of GBP 9.50 per hour [35].

#### 2.3.2. The MW Programme Outputs

The MW programme consisted of one four-hour taster day followed by an eight-week programme of one five-hour session per week. Each five-hour session included two nature-based group activities, one in the morning and one in the afternoon, a shared lunch, plus mindfulness and complementation techniques in natural settings. Nature-based activities included a range of traditional green crafts including wool weaving, wood whittling, willow weaving, and willow-structure creation (e.g., natural compost bins and living willow arches) and therapeutic horticulture activities such as planting and coppicing. Group nature-based activities and the shared lunch provided participants time for socialisation throughout the programme, enabling the development of connections and friendships.

#### 2.3.3. The MW Programme Outcomes

Stakeholder outcomes identified to be most important in this evaluation were health and well-being-related outcomes for MW programme participants, including improved overall well-being, improved mental health, increased confidence, increased social connection, and a reduction in GP service use.

### 2.4. Evidencing and Valuing Outcomes

#### 2.4.1. Evidencing Outcomes

A mixed-method approach was used to collect quantitative and qualitative information on MW programme participants’ self-reported overall well-being, mental health, self-confidence, social connection, and GP service utilisation. Questionnaires with quantitative outcome measures were completed by MW programme participants at baseline and nine-week follow-up. Participants were also invited to undertake a semi-structured telephone interview following completion of the MW programme. Informed consent was obtained from participants for baseline and follow-up questionnaires and interviews before the MW programme started.

Well-being valuation was used to assign financial proxy values to quantitative outcome data collected via questionnaires. Well-being valuation offers a consistent and robust method for estimating the financial value of health and well-being-related outcomes that do not have a market value [27]. A well-being valuation of expected participant outcomes was undertaken using two social value calculators: the HACT Social Value Calculator v.4 derived from the Social Value Bank (SVB) [36], and the Mental Health Social Value Calculator derived from the Short Warwick–Edinburgh Mental Well-being Scale (SWEMWBS) [37]. These calculators are applied separately to self-reported participant outcomes to estimate a range of SROI ratios as a form of embedded sensitivity analysis.

The HACT Social Value Calculator v.4 was used to apply financial proxy values to the expected outcomes of improved overall well-being, increased social connection, and increased confidence, measured using the ICEpop CAPability measure for Adults (ICECAP-A) instrument, SWEMWBS Question 6 (‘I’ve been feeling close to other people’), and General Self-Efficacy Scale (GSES), respectively (Figure 1). The HACT Mental Health Social Value calculator v.1 was used to value improvements in mental health measured using the full SWEMWBS.

The SWEMWBS is a short version of the Warwick–Edinburgh Mental Well-being Scale (WEMWBS), which is validated and reliable for a range of UK populations and settings [38]. The scale contains seven statements with five response groupings linked with characteristics of positive mental health and has a score range of 7 to 35, with 7 reflecting very poor mental health and 35 reflecting excellent mental health. SWEMWBS Question 6 focuses on social connection (‘I’ve been feeling close to other people’).

The ICECAP-A instrument is a validated capability-based 5-item health and well-being measure for the general adult (aged over 18 years) population [39,40]. It encompasses a broad definition of health and well-being by including five attributes of well-being that were found to be important to adults in the UK. These attributes include: (i) feeling settled and secure; (ii) love, friendship, and support; (iii) being independent; (iv) achievement and progress; and (v) enjoyment and pleasure. Each attribute has four response levels, each associated with a pre-defined tariff value. A total tariff value reflecting an overall well-being-related quality of life state is calculated summing the individual tariff values that correspond with the response levels selected across all five attributes. These total tariff values can range from −0.001 to 1, with 1 reflecting full capability or well-being.

The GSES is 10-item measure of self-efficacy and confidence. It assesses the strength of an individual’s belief in their ability to effectively respond to novel or challenging situations, overcoming any associated obstacles or setbacks [41]. Overall scores on the GSES can range from 10 to 40, with higher scores indicating greater self-efficacy and confidence.

In addition to the above quantitative outcome measures, a bespoke CSRI form was included in baseline and follow-up questionnaires to measure participants’ GP service use [42]. The CSRI asked participants to recall the number of times they had visited the GP for two different time periods: eight weeks before they started the MW programme and eight weeks during the programme.

Questionnaire response rates were explored to understand the acceptability and feasibility of using the above measures for people with mild-to-moderate mental health conditions. Questionnaire data were analysed to determine the number of participants who improved, stayed the same, or worsened for each outcome between baseline and follow-up.

Follow-up interviews took place over the phone at a time that suited the interviewee and lasted up to 45 min. Interviews were audio-recorded and transcribed with permission from the interviewee. The interviews aimed to collect further, qualitative evidence of the changes participants experienced because of the MW programme, including which outcomes were most important to them. Participants were also asked how likely they would recommend the MW programme to others, and were invited to provide any general feedback and suggestions regarding the programme and its future development. Thematic narrative analysis was conducted on all data gathered from interviews to categorise the findings into key emerging themes [43,44].

#### 2.4.2. Valuing Outcomes

Three alternative well-being valuation approaches were applied to the reported participant outcomes of improved overall well-being, improved mental health, increased social connection, and increased confidence to estimate a robust range of social values. Two of these well-being valuation approaches used the Social Value Calculator to apply financial proxy values from the HACT Social Value Bank (SVB) to (i) improved overall well-being, and (ii) an increased sense of belonging and increased confidence, separately. The HACT Mental Health Social Value Bank (MHSVB) is a framework that assigns monetary values to over 100 distinct social outcomes, employing a well-being valuation methodology. This robust methodology draws on statistical analysis of thousands of data points from the British Household Panel Survey and other large-scale British surveys. For instance, based on this survey data, ‘Good overall health’ is assigned a value of GBP 20,141. These assigned financial proxies are also known as ‘social values’ or ‘monetary values’. These three participant outcomes were separated and valued in this way because of the known relationship and lack of independence between well-being, social connection [45], and self-confidence [46].

The SVB financial proxy of GBP 20,141 for ‘Good overall health’ was applied to the participant outcome of improved overall well-being based on a 10% or more improvement in ICECAP-A scores between baseline and follow-up (i.e., if six participants report a 10% or more improvement in ICECAP-A score, a social value of 6 × GBP 20,141 would be included in the well-being valuation, following a similar approach to Hartfiel et al. 2023 [47]. Similarly, SVB financial proxy values of GBP 13,080 for ‘High Confidence (adult)’ and GBP 3753 for ‘Feel belonging to neighbourhood’ were applied to the participant outcomes of increased sense of belonging (social connection) and increased confidence, based on a 10% or more improvement in SWEMWBS Q6 score and overall GSES scores, respectively, between baseline and follow-up (Table 1).

The third well-being valuation approach used the Mental Health Social Value Calculator to apply financial proxy values derived directly from overall SWEMWBS scores reported by each participant at baseline and follow-up, following the methodology of Trotter and Rallings Adams (2017) to estimate the mental health-related social value generated by the MW programme (Table 1) [37].

Finally, to estimate the social value generated for NHS Wales by the MW programme, the unit cost of a GP consultation in 2021/2022 (GBP 41 per appointment, including GP qualification costs [48] was used to estimate the social value generated by the change in number of GP appointments eight weeks before the programme and eight weeks during the programme (Table 1).

### 2.5. Establishing Impact

To ensure a robust estimation of net social value using the Social Value Calculator, participant questionnaires included specific questions regarding deadweight, attribution, and displacement to help determine the extent to which the MW programme impacted reported participants outcomes. In SROI methodology, these aspects of understanding the extent of an intervention’s impact are applied to reduce bias and the risk of over-claiming benefits.

#### 2.5.1. Deadweight

Deadweight acknowledges that there is likely to be a proportion of the reported participant outcomes that could have happened even if participants did not attend the MW programme. Deadweight was estimated by including a specific question in the follow-up questionnaire that asked participants to consider how many of the changes they had experienced would have happened regardless of their participation in the programme.

#### 2.5.2. Attribution

Attribution refers to the proportion of reported participant outcomes that can be directly attributed to the programme. Attribution was estimated by including a specific question in the follow-up questionnaire that asked participants to consider how many of the changes they had experienced would have happened because of their participation in the programme.

#### 2.5.3. Displacement

Displacement considers whether participants had to give up any other activities that could have contributed to their health and well-being to attend the MW programme. Displacement aims to estimate the foregone benefits or opportunities resulting from the intervention, or the ‘opportunity cost’ of the intervention to participants. To estimate displacement the follow-up questionnaire asked participants how much they had to give up other activities that could have benefitted them due to attending the programme.

#### 2.5.4. Mental Health Social Value Calculator

The percentages assigned to deadweight, attribution and displacement are estimates based on self-report by participants who completed baseline and follow-up questionnaires. To reduce the risk of over-claiming benefit when using the Mental Health Social Value Calculator to estimate net social value, a 27% standard deadweight percentage for was subtracted from the total SWEMWBS-estimated values [37].

### 2.6. Calculating the SROI Ratio

SROI ratios were calculated to compare the estimated social value of relevant outcomes per participant with total MW programme costs per participant. These ratios express the MW programme’s value for money in terms of the amount of social value created per GBP 1 invested (Equation (1)).

(1) $$SROI\;ratio=\frac{Social\;value\;of\;MW\;programme\;outcomes\;per\;participant}{MW\;programme\;costs\;per\;participant}$$

### 2.7. Social Value Forecast

The social cost–benefit evidence generated by the SROI evaluation were incorporated into a hypothetical SROI forecast of the annual net social value estimated to be generated by the MW programme based on several planned changes to programme delivery and an assumption that 12 MW programmes could be delivered over a 12-month period (3 programmes delivered concurrently each quarter). These expected changes were based on Fathom’s commitment to improving programme delivery, increasing the frequency of programme delivery, and to reducing participant costs.

The total forecast cost of delivering the updated MW programme over a 12-month period presented here includes recalculated costs of running the MW programme based on Fathom’s planned changes plus estimated 20% organisational overheads for Fathom (assumed to be 20% of total programme running costs). These costs and their underlying assumptions are outlined below.

#### 2.7.1. Organisational Overheads

Organisational overheads at a rate of up to 20% of total programme cost were calculated and applied to the SROI forecast to reflect an estimated sustainable future cost of the MW programme over the next three years. Organisational overheads for charities typically include the costs of governance and staff salaries and expenses for management, administration, and fundraising [49,50,51]. Moderate not-for-profit overheads are considered to fall in the range of 20% to 25% [52,53], for example, the UK Civil Society Almanac 2021 indicates that average overhead costs (including spend on governance, grants and fundraising activity) for micro to small voluntary sector organisations in the UK were 21% [54].

In line with the approach taken by Portillo and Stinn (2018), an estimated organisational overhead of 20% is applied to estimate future governance, management, administrative and fundraising/grant capture inputs into the MW programme to forecast the potential social value of the MW programme over a 12-month period [52]. The overheads outlined below are considered essential for enabling Fathom to operate effectively and achieve financial sustainability [29,51,52], and are applied to reduce the risk of over-estimating the potential social value the programme can generate.

**Board of Trustees:** Fathom expects to hold three out of four one-hour annual meetings online and one two-and-a-half hour meeting per year face-to-face (5.5 h of meetings in total per annum). The estimated cost of room hire and catering is GBP 150 per annum. To ensure transparency in the associated Trustee costs this cost is divided between three programmes delivered by Fathom (MW, DareGale, Folk School) and an attributable cost of GBP 50 per annum in Trustee specific overheads for the MW programme. Trustee salary and hourly rate estimates are presented in Table S1. The total estimated volunteering opportunity cost for the board of Trustee’s is GBP 195.81 per meeting and GBP 1076.96 per annum based on 5.5 h of meetings in total. When divided between three Fathom programmes, an attributable cost GBP 358.99 per annum in Trustee-specific opportunity costs for MW programme delivery over 12 months is estimated and applied to the SROI forecast.

**Programme Development Board:** Fathom intends to establish a Programme Development Board (PDB) to advise on programme design, delivery, and development. The Board is expected to comprise eight individuals representing a range of relevant sectors and organisations and meet four times a year, with two one-hour meetings held online and two, two-and-a-half hour meetings held face-to-face (seven hours of meetings per annum). Catering and travel costs is estimated to be GBP 15 per member per meeting, giving a total expected cost of GBP 240 per annum for two face-to-face meetings and a MW specific overhead cost of GBP 80 per annum (GBP 240/3 = GBP 80). PDB member salary and hourly rate estimates were calculated using average UK salary estimates from payscale.com [31] and glassdoor.co.uk [54]. The total estimated volunteer opportunity cost for the PDB is GBP 207 per hour and GBP 1449 per annum based on seven hours of meetings in total (Table S2). An attributable cost of GBP 483 per annum in PDB-specific opportunity costs for MW programme delivery over 12 months is applied in the SROI forecast (GBP 1449/3 = GBP 483).

**Programme management:** Three-hours of Charity Director time per day of the nine-week MW programme is expected for day-to-day management, giving at total of 324 h management time for 12 MW programmes delivered over a 12-month period. 324 h of management time at GBP 23.36 per hour gives a total cost of GBP 7568.20 in management overheads over a 12-month period.

**Programme administration:** Based on the six-month MW programme piloted here, an average of 27 h of Administrative Assistant time is expected to be required for the administration of each MW programme, equating to 324 h of administration time for the delivery of 12 MW programmes over a 12-month period. An estimated hourly rate of GBP 9.93 per hour is applied based on an average Administrative Assistant salary of GBP 18,843 per annum sourced from payscale.com [31]. 324 h of administration time at GBP 9.93 per hour gives total cost of GBP 3217.46 in administrative overhead support required for delivery of 12 MW programmes.

**Charity Fundraising:** To ensure long-term sustainability of the charity, Fathom will seek to raise funds to support charity activities. Fathom conservatively estimates that a total of 70 h per annum will be required to be spent on grant writing and fundraising endeavours. Applying the conservative estimated time of 70 h of fundraising activity at a rate of GBP 23.56 per hour provides a total cost of GBP 1649.20 towards fundraising activity linked to the MW programme.

**Continued Professional Development:** Fathom expects to spend GBP 250 on safeguarding and other relevant training for each craft and therapeutic horticulture session instructor every two years, across a team of five, with an associated cost of GBP 625 per annum. Continual Professional Development (CPD) costs were divided between three Fathom programmes to calculate a specific overhead cost of GBP 208.33 per annum towards CPD training for the MW programme. Fathom also aspires to organise at least one ‘away day’ per year to support instructor development and well-being, with an estimated associated cost of GBP 400 per day. When split between the three Fathom programmes, would equates to an additional overhead cost of GBP 133.33 per annum.

**The Fathom Trust Website:** A website is considered essential for establishing an online presence and supporting communication and marketing of Fathom programmes to a wider audience. Website hosting costs are GBP 6 per month, giving a total cost of GBP 72 per annum. This annual cost was divided between three Fathom programmes to give a MW programme-specific overhead cost of GBP 24 per annum.

#### 2.7.2. Direct MW Programme Costs

The assumptions underlying the recalculated costs of running the MW programme over 12 months are presented below.

**Site hire:** Fathom is committed to widening the range of locations where the MW programme is available. The expansion of venues is expected to include local, rural village halls and community centres in addition to the current location at Llanfellte Farm. Village hall hire across Powys ranges from GBP 30 to GBP 75 per day and rent for Llanfellte Farm continues to be estimated at GBP 100 per day. A mid-point cost of GBP 75 for site hire or rent per day was used in the 12-month SROI forecast. The estimated total cost of site hire and rent for 12 MW programmes over a 12-month period is GBP 8100.

**Catering:** Lunch and light refreshments were estimated at a cost of GBP 8.00 per person, increased from GBP 7.50 per person used in the SROI evaluation to consider inflation of food prices. The total cost of catering for 12 MW programmes over a 12-month period is GBP 17,280.

**Other direct costs:** All other direct costs of the MW programme for the SROI forecast were estimated based on the same assumptions used in the SROI evaluation.

## 3. Results

### 3.1. MW Programme Input Costs

The value of MW programme inputs between October 2021 to March 2022 were estimated to be GBP 9843 per programme. Costs included craft and horticulture sessions, and management and administration, valued at GBP 4750 and GBP 1474.20 per programme, respectively. The total costs per participant were estimated at GBP 1312 per person per programme (Table 2).

### 3.2. Stakeholder Outcomes

A total of 20 participants enrolled onto two MW programmes during this pilot evaluation, and 19 out of the 20 participants opted to complete a baseline questionnaire at the start of their nine-week programme. Further, 4 of these participants dropped out of the MW programme (i.e., stopped attending sessions), and 15 participants completed the programme and a follow-up questionnaire, giving a complete case response rate of 75%. The reasons for participants dropping out of the programme are unknown and the collection of this data was beyond the scope of this pilot study. Only complete cases are included in the SROI analyses reported here. Baseline demographic characteristics are presented in Table 3.

Response rates for all health and well-being outcome measures were high, ranging from 93% for ICECAP-A (14 out of 15 participants) to 100% for all other outcome measures, including SWEMWBS, GSES (Table 4) and the bespoke CSRI (Table 5). Due to missing data, one participant’s ICECAP-A results could not be included.

A comparison of mean participant outcome measure responses at baseline and follow-up indicates overall positive change for participants across all outcome measures (Table 4). The number of MW programme participants reporting a ≥10%, ≥25% and ≥50% improvement in outcome measure scores between baseline and follow-up are presented in Table 5.

With respect to NHS Wales outcomes and the number of participant appointments with the GP over an eight-week period, a 40% reduction in GP appointments was reported at follow-up compared to baseline (Table 6).

### 3.3. Establishing Impact

Questionnaire results provided an estimated average deadweight of 15% (i.e., 15% of the change participants experienced since the start of the programme would have happened anyway), an estimated average attribution of 18% (i.e., 18% of the change participants had experienced would have happened without the programme), and an estimated average displacement of 13% (i.e., participants indicated that they had to forego 13% of other beneficial activities in order to attend the programme).

#### 3.3.1. Well-Being Valuation Using the Social Value Calculator

When the above estimates for deadweight, attribution and displacement are applied to the financial proxy values for improved overall well-being, increased social connection and increased confidence from the HACT SBV (Table 1), and number of participants that reported a 10% or more improvement in outcome measure scores (Table 5), the net social value for MW participants is estimated to be GBP 6107 per participant for improved overall well-being (Table 7) and GBP 4538 per participant for increased social connection and increased confidence (Table 8).

#### 3.3.2. Well-Being Valuation Using the Mental Health Social Value Calculator

When applying the HACT Mental Health Value Calculator, the total social value generated by the MW programme is estimated to be GBP 88,155, based on the change in participant SWEMWBS scores between baseline and follow-up. Following the subtraction of the 27% standard deadweight, the estimated net mental health-related social value experienced by MW programme participants was GBP 4290 per participant (Table 9). 

#### 3.3.3. Valuing Outcomes from the CSRI Questionnaire

Applying the GBP 41 unit cost of a GP appointment (Table 1) to the 40% reduction in GP appointments reported by participants (Table 6) resulted in an estimated average cost saving of GBP 32.80 per participant.

### 3.4. Qualitative Results from Semi-Structured Interviews

Semi-structured telephone interviews were conducted with 12 of the 15 participants that completed the MW programme (80% interview response rate). The qualitative information collected via these interviews support the quantitative results reported above. Three key themes of improved mental well-being, increased sense of belonging, and higher confidence emerged from the thematic analysis of interview responses, illustrated by the examples of participant quotations below:

Improved mental health

- “I found it really relaxing just listening to nature, closing my eyes and I felt really calm…More relaxed, more calm and at peace.”
- “I’ve gone from being quite an anxious person to somebody who kind of looks for things to relieve anxiety when it comes and I feel more able to do that.”
- “I’m definitely feeling less anxious.”

Increased social connection

- “I felt connected and suppose I felt that I wasn’t on my own. I had people there that I felt safe with.”
- “We were able to grow together…craft together and work together as a team.”
- “Getting out and talking to people…the social aspect was the biggest thing for me that helped.”

Increased confidence

- “There’s a belief in myself that I have got more to offer.”
- “We have got the skills now to move forward on our own.”
- “The sense of achievement when you actually produce something…and getting over the difficulties as well and not giving up—stepping away and coming back and having the support of the person next to you.”

A very strong theme throughout the interviews was the sense of belonging and connection participants had experienced because of the programme. All 12 participants noted that connection with group members and session instructors, and appreciation of a warm, welcoming, non-judgmental, safe, and supportive community was key to their positive experience of the programme and its benefits. Nine out of twelve interviewees stated that they had experienced an increased sense of social connection and self-confidence, and seven out of twelve interviewees reported a noticeable improvement in their mental health.

Learning new skills (in crafting, horticulture, and managing personal mental health) was also highlighted by many participants as key elements and outcomes of the programme. Two participants mentioned the physical aspect of the programme and an improvement in physical health, and two participants acknowledged that the MW programme had helped them return to work and find a new job, respectively, suggesting that the MW programme has potential wider health, social, and economic impact beyond mental health and well-being. 100% of participants interviewed (*n* = 12) stated they would be likely (*n* = 1, 8.3%) or very likely (*n* = 11, 91.7%) to recommend the MW programme to others.

### 3.5. Calculating the SROI Ratio

The SROI ratio is calculated by dividing the total value of MW programme inputs by the net social value of reported participant outcomes to estimate the amount of social value generated per participant for every GBP 1 invested.

Three different SROI ratios calculated using three alternative well-being valuation approaches combined with the cost savings resulting from the reduction in GP appointments indicate that the MW programme generates social value in the range of GBP 3.30 to GBP 4.70 for every GBP 1 invested (Table 10). However, there is some uncertainty in this range of SROI ratios due to the small sample size and lack of a control group.

### 3.6. SROI Forecast

The social cost–benefit evidence from this SROI evaluation suggests that the social value generated per participant could be improved by reducing programme costs per participant. To achieve this, Fathom commits to working more closely with the local primary care cluster and relevant third sector organisations to raise awareness of the MW programme and increasing the number of participant referrals per programme by 20%, expanding the number of venues where people can access the MW programme and reducing site rent costs, investing in administrative support to reduce demands on Charity Founder and Director time, and providing safeguarding and other relevant training for craft and therapeutic horticulture session instructors to remove the need for a Community Support Worker to be present. Fathom also aspires to deliver 12 MW programmes per annum, with three programmes running concurrently each quarter. The above commitments and aspirations to develop the MW programme were applied and extrapolated using simple methodology and assumptions to forecast the hypothetical social value that could be generated for a 12-month delivery of the MW programme in the future.

#### 3.6.1. Forecast Costs

Fathom Trust organisational overheads were estimated to be GBP 13,758 per annum (Table 11). When considering only the direct costs of delivering 12 MW programmes over a 12-month period, these were estimated to be GBP 93,360 in per annum and GBP 648 per participant (Table 12). Projected 20% organisational overheads of GBP 21,424 were estimated by calculating 20% of direct programme costs (GBP 93,360) and adding these to the initial expected 12-month organisational overhead costs of GBP 13,758 per annum (Table 11) (i.e., 20% of 93,360 + GBP 13,758 = 20% of GBP 107,118 = GBP 21,424)). The projected 20% organisation overhead of GBP 21,424 was combined with direct programme costs of GBP 93,360 to forecast total estimated sustainable costs of GBP 114,784 per annum and GBP 797 per participant to deliver 12 MW programmes over a 12-month period (Table 12).

#### 3.6.2. Forecast SROI Ratios

Forecasting SROI ratios using the above extrapolated hypothetical costs suggests that 12 MW programmes delivered over a 12-month period may have the potential to generate social value in the range of GBP 5.40 to GBP 7.70 per every GBP 1 invested (Table 13).

## 4. Discussion

This pilot SROI evaluation and forecast of the MW programme explored the acceptability and feasibility of several validated quantitative health and well-being outcome measures and their application to SROI evaluation and forecast methodology to understand the social cost–benefit and social value of the MW NBSP programme. The high response rates for all health and well-being outcome measures used (93% to 100%) suggest that these measures are acceptable to people with mild-to-moderate mental health conditions and are feasible to use in a larger scale study of NBSP programme. This is the first published SROI that we have seen to use ICECAP-A as a validated, outcome measure for assessing overall health and well-being. This capability-based measure is considered more appropriate for assessing health and well-being impacts of programmes designed to support mental health, compared to measures such as the EQ-5D, due to its focus on broader determinants beyond physical aspects of health [55,56].

The questionnaire and interview data demonstrate that the MW programme is a positive supportive well-being intervention for people with long-term mild-to-moderate mental health conditions. Participants reported improved overall well-being, improved mental health, increased social connection, and increased confidence. No significant negative outcomes were reported, and 11 of 12 participants interviewed at follow-up stated they would ‘very likely’ recommend the MW programme to others. The positive changes experienced by participants during the MW programme may have had an impact on their health service use, implied by a 40% reduction in the number of GP visits over an eight-week period reported at the end of the programme compared to baseline. The CSRI required participants to recall their number of GP visits over an eight-week period, which may be subject to recall bias.

Applying well-being valuation to the above participant reported outcomes indicates that the MW programme generates a positive SROI ranging from GBP 3.30 to GBP 4.70 for every GBP 1 invested. These SROI ratios are comparable to other NBSPs and NBIs, such as the Green Gym which involves volunteer participation in local conservation activities, including habitat management and creation. An SROI evaluation of the Green Gym identified that volunteers experienced improved physical health, reduced social isolation, and increased personal well-being, and estimated an SROI of GBP 4.02 for every GBP 1 invested [57]. A Community Garden Project at Gorgie City Farm generated a range of volunteer outcomes, including improved confidence, better mental health, being more physically active, and spending more time with friends, resulting in GBP 3.56 of social value generated for every GBP 1 invested in the project [58]. Finally, a 12-week outdoor walking and climbing green social prescribing programme, ‘Opening Doors to the Outdoors’, was found to generate social value in the range of GBP 4.90 to GBP 5.36 per GBP 1 invested [20].

A hypothetical SROI forecast of the potential social value that could be generated by the MW programme suggests that making simple changes such as increasing the number of participants attending each programme from 10 to 12, employing an Administrative Assistant, and providing appropriate safeguarding and other supportive training to staff, could lead to an increased ROI of GBP 5.40 to GBP 7.70 for every GBP 1 invested.

Whilst there are examples of SROI evaluations and forecasts applied separately to NBIs, we cannot find published examples of studies that have combined SROI evaluation and forecasting to inform real-world implementation. Bagnall et al. (2019) estimated a SROI GBP 6.88 for every GBP 1 invested in The Wildlife Trusts programmes designed to improve mental, physical and social well-being [59], indicating comparable social value to our forecast for the MW programme.

### 4.1. Strengths

The mixed-method SROI methodology employed in this innovative combined evaluation and forecast pilot uses quantitative and qualitative data collection to provide a broader evidence-base for well-being valuation and social value estimation compared to a single form of data collection. This broad approach is particularly important when identifying and evaluating the impacts of complex interventions from a stakeholder perspective in real-world contexts. The use of the well-established HACT Social Value Bank, Social Value Calculator, Mental Health Social Value Calculator, and three alternative well-being valuation approaches as a form of embedded sensitivity analysis, gives confidence in the SROI results.

The high health and well-being outcome measure response rates give confidence that the chosen outcome measures were suitable for the purpose of this pilot SROI evaluation and forecast, giving confidence in the pilot results, and are likely to be feasible for use in larger scale NBSP and NBIs studies. The high response rate for ICECAP-A is particularly encouraging and insightful, given that this capability outcome measure has not been used in other published SROI evaluations or forecasts of NBSP to date, despite its value for assessing overall health and well-being compared to measures like the EQ-5D that tend to have a narrower focus on the physical aspects of health and well-being.

### 4.2. Limitations

This SROI evaluation and forecast focuses on two stakeholder groups, (i) MW programme participants and (ii) NHS Wales. The MW programme is expected to have impacts for other stakeholders, such as other third sector organisations like Brecon & District MIND that refer participants to the MW programme, and MW programme participant family members and friends who may indirectly benefit from the MW programme due to positive changes experienced by participants. Collection of this data was outside the scope of the six-month pilot and may result in an underestimation of the total social value generated by the MW programme.

The small-scale case study approach used in this pilot evaluation had critical constraints, including a small sample size of 15 participants and the absence of a comparator intervention or control group. The small sample size means that the ability to generalise participant reported outcomes and estimated social value to the wider population is limited. The lack of a comparator intervention or control group hampers the exploration of causality and our understanding of the extent to which reported improvements in participant outcomes are a direct result of the MW programme, especially given the complexity of well-being and mental health outcomes. Participant outcomes and impact adjustments (deadweight, attribution and displacement) are based on participant self-report and may therefore be subject to bias. We also acknowledge that our calculation of volunteer opportunity costs using the National Living Wage is a simple assumption that may not reflect the true social value of volunteer time, leading to further uncertainty in our socal value estimates.

Whilst the application of deadweight, attribution, and displacement in SROI methodology helps to mitigate against over-claiming the programme’s positive impact [60], the above limitations mean that the SROI of the MW programme at scale across the total population is uncertain and may be lower than the SROI estimated here.

Our pilot forecast SROI is based on the social cost–benefit evidence provided by the SROI evaluation, subject to its own limitations (see above), plus simple forecast methodology and assumptions due to the time restrictions of this small-scale 6-month pilot study. Our approach and assumptions may introduce further bias or uncertainty in our forecast estimates. We acknowledge, for example, the implicit assumption that social value generated by the MW programme will not change when extrapolated over a 12-month period (i.e., we have not accounted for diminishing returns). We also acknowledge that whilst our calculation of overhead costs is based on ~20% estimates reported in existing literature, this assumption may not be accurate and may lead to bias. Finally, we did not have the time or scope in this study to consider potential implementation barriers to Fathom’s aspirations and intentions to develop the programme and expand its reach. Our forecast estimates are therefore based on simple linear scalability of the programme. We emphasise the hypothetical nature of our initial pilot forecast and present our approach and assumptions as a useful starting point to be refined and built upon in the future.

### 4.3. Recommendations for Future Research

Recommendations for future research include increasing the sample size to enhance representativeness of the findings. The inclusion of a control group is important for future research to accurately attribute observed changes to the NBI. Furthermore, analyses could incorporate measures of chronicity of symptoms and disability to provide a more nuanced understanding of the long-term effects and benefits for different sub-populations. It is acknowledged that larger-scale and longer-term evaluations are required to better understand the generalisability of NBSP and NBI impacts to support integration into policy and practice. We believe SROI underpinned by strong study design, alongside RCT or natural experiments [61] is a useful methodological tool in the box for evaluating complex interventions that have the potential to generate a range of outcomes across different groups of stakeholders.

## 5. Conclusions

This health economics-informed SROI of the pilot Making Well programme demonstrates the role of SROI evaluation and forecasting in enabling third sector organisations to understand, monitor, and optimise SROI, which can be used to secure sustainable funding into the future and better support the health and well-being of vulnerable groups in the context of diminishing resources, and in this case the use of NBIs.

Despite the small sample size and lack of a control group, our results contribute to the evidence-base for the effectiveness and social return on investment of NBSPs as a therapeutic intervention for improving health and well-being [62,63] and provides an example of the use of health economic well-being outcome measures such as ICECAP-A and CSRIs in social value analysis.

## Figures and Tables

**Table 1:** Well-being valuation approaches and outcomes used to calculate the social value generated by the MW programme, including how outcomes were defined and valued.

Well-Being Valuation Approach	Outcome	Outcome Indicator	Financial Proxy	Value (GBP)
Social Value Calculator applied to a general well-being outcome	Improved overall well-being	Improvement of 10% or more in ICECAP-A score between baseline and follow-up. Total tariff values calculated using this measure can range from −0.001 to 1, with 1 reflecting full capability or well-being.	HACT Social Value Calculator v4: Good overall health	20,141
Social Value Calculator applied to two specific well-being outcomes	Increased confidence	Improvement of 10% or more in General Self-Efficacy Scale (GSES) score between baseline and follow-up.	HACT Social Value Calculator v4: High confidence (adult)	13,080
	Increased social connection	Improvement of 10% or more in SWEMWBS Q6 score (‘I’ve been feeling close to other people’) between baseline and follow-up.	HACT Social Value Calculator v4: Feel belonging to neighbourhood	3753
Mental Health Social Value Calculator	Improved mental health	Improvement in SWEMWBS score between baseline and follow-up. Total scores on this scale can range from 7 to 35.	HACT Mental Health Social Value Calculator v1.	Various, depending on SWEMWBS score
National Unit Cost	Reduction in GP appointments	Difference in GP appointments 8 weeks before the programme and 8-weeks during the programme.	Unit cost of a GP consultation 2021/2022 [43]	41

**Table 2:** Estimated Making Well programme costs during the six-month pilot, including cost per participant.

Cost Category	Cost Description	Cost Source	Average Cost per Day (GBP)	Nine-Week Programme Costs (GBP)	Six-Month Pilot Costs (GBP)	Cost per Participant (GBP) (*n* = 15)
The Fathom Trust board of Trustees (Governance)	Six months of board meetings	The Fathom Trust, payscale.com [31]	GBP 7.25 (GBP 65.25/9)	GBP 65.25 (GBP 130.50/2)	GBP 130.50	GBP 8.70
Management and administration	Charity Director estimated average hourly rate GBP 23.40 × 7 h	payscale.com [31]	GBP 163.80	GBP 1474.20	GBP 2948.40	GBP 196.56
Site rent	Daily rent, including utilities, for Llanfellte Farm, Brecon.	The Fathom Trust	GBP 100	GBP 900	GBP 1800	GBP 120
Craft and horticulture sessions (Staff, equipment, materials)	GBP 250 per craft, 3 crafts on taster day, 2 crafts per day thereafter	The Fathom Trust	GBP 527.78 (GBP 4750/9)	GBP 4750	GBP 9500	GBP 633.30
Outdoor heating	Fire bowls and wood	The Fathom Trust	GBP 18.89 (GBP 170/9)	GBP 170	GBP 340	GBP 22.66
Catering for participants, staff and volunteers	GBP 7.50 per person Catering for participants, staff and volunteers	The Fathom Trust	GBP 120	GBP 1080	GBP 2160	GBP 144
Support staff	Community Support Worker at GBP 10.85 per h × 5 h per day	payscale.com [31]	GBP 61	GBP 549	GBP 1098	GBP 73.20
Volunteer time (10 h per session)	(GBP 9.50 × 10 h)	gov.uk/national-minimum-wage-rates [34]	GBP 95	GBP 855	GBP 1710	GBP 114
TOTAL (rounded to the nearest pound):			GBP 1094	GBP 9843	GBP 19,687	GBP 1312

**Table 3:** Baseline demographic characteristic of MW programme participants who completed baseline and follow-up questionnaires.

Demographic Characteristics	Participants (*n* = 15)
**Age (years)**	
Mean ± Standard deviation	46.53 ± 11.46
Minimum–Maximum	29–63
**Gender**	
Female, *n* (%) Male, *n* (%)	8 (53%) 7 (47%)
**Employment status**	
Employed, *n* (%)	4 (27%)
Unemployed, *n* (%)	9 (60%)
Other, *n* (%)	2 (13%)

**Table 4:** MW participant outcome measures at baseline and follow-up.

MW Participant Outcome	Outcome Measure	No. of Respondents	Baseline Mean ± SD	Follow-Up Mean ± SD	Mean Difference #
Overall well-being	ICECAP-A	14	0.611 ± 0.200	0.711 ± 0.167	0.100
Mental health	SWEMWBS	15	20.07 ± 6.86	24.27 ± 4.30	4.2
Social connection	SWEMWBS Q6	15	2.60 ± 1.06	3.47 ± 0.64	0.87
Self-confidence	GSES	15	23.93 ± 6.03	26.7 ± 5.3	2.77

# Mean difference is calculated by subtracting baseline mean values from follow-up mean values. A positive mean difference suggests an overall positive effect of the Making Well programme effect. Statistical significance is not assessed due a lack of a control group for comparison and small sample sizes.

**Table 5:** Number of Making Well programme participants reporting a ≥10%, ≥25% and ≥50% improvement in outcome measure scores between at baseline and follow-up.

Outcome	Outcome Measure	No. of Respondents	No. of Participants Reporting a ≥10%, ≥25% and ≥50% Improvement in Outcome Scores		
			≥10% Improvement *n* (%)	≥25% Improvement *n* (%)	≥50% Improvement *n* (%)
Overall well-being	ICECAP-A	14	7 (50.0)	6 (42.8)	2 (14.3)
Mental health	SWEMWBS	15	8 (53.3)	6 (40.0)	4 (26.7)
Social connection	SWEMWBS Q6	15	9 (60.0)	9 (60.0)	8 (53.3)
Self-confidence	GSES	15	6 (40.0)	4 (26.7)	2 (13.3)

**Table 6:** Change in the total number of Making Well programme participant visits to the GP over an eight-week period between baseline and follow-up.

Participant Outcome	Outcome Measure	n	No. of Appointments at Baseline	No. of Appointments at Follow-Up	Percentage Difference
Change in number participant appointments with GP	GP appointments reported in participant CSRI	15	30	18	−40% (−12 appointments)

**Table 7:** Social value for MW programme participants estimated using the Social Value Calculator applied to improved overall well-being and adjusted for deadweight, attribution and displacement.

Outcome	No. of Participants Experiencing Outcome	Value	Total Value	Mean Deadweight	Mean Attribution	Mean Displacement	Net Social Value	Net Social Value per Participant
Improved overall well-being	7 out of 14 participants reported a 10% or more improvement in ICECAP-A score	GBP 20,141	GBP 140,987	15%	18%	13%	GBP 85,493	GBP 6107
**Social value generated by the Making Well programme (total and per participant):**							GBP 85,493	GBP 6107

**Table 8:** Social value for Making Well programme participants estimated using the Social Value Calculator applied to increased social connection and increased confidence and adjusted for deadweight, attribution and displacement.

Outcome	No. of Participants Experiencing Outcome	Financial Value	Total Financial Value	Mean Deadweight	Mean Attribution	Mean Displacement	Net Social Value	Net Social Value per Participant
Increased confidence	6 out of 15 participants reported a 10% or more improvement in GSES score	GBP 13,080	GBP 78,480	15%	18%	13%	GBP 47,589	GBP 3173
Increased social connection	9 out of 15 participants reported a 10% or more improvement in SWEBWMS Q6 score	GBP 3753	GBP 33,777	15%	18%	13%	GBP 20,482	GBP 1365
**Social value generated by the Making Well programme (total and per participant):**							GBP 68,071	GBP 4538

**Table 9:** Net mental health-related social value generated for Making Well programme participants, calculated using Mental Health Social Value Calculator.

Outcome	N	Total Social Value at Baseline	Total Social Value at Follow-Up	Difference in Social Value	Deadweight	Net Social Value	Net Social Value per Participant
Improved mental health	15	GBP 242,678	GBP 330,833	GBP 88,155	27%	GBP 64,353	GBP 4290
**Social value generated by the Making Well programme (total and per participant):**						GBP 64,353	GBP 4290

**Table 10:** Social value generated by the Making Well programme, calculated for the six-month Making Well pilot using three different well-being valuation approaches.

	Well-Being Valuation Approach		
	Social Value Calculator Applied to Improved Overall Well-being	Social Value Calculator Applied to Increased Social Connection and Increased Confidence	Mental Health Social Value Calculator Applied to SWEMWBS Scores
Outcomes	Improved overall well-being	Higher confidence and Increased sense of belonging (social connection)	Improved mental health
Well-being-related Social Value per participant	GBP 6107	GBP 4538	GBP 4290
NHS Cost-saving per participant	GBP 33	GBP 33	GBP 33
Total Social Value per participant	GBP 6140	GBP 4571	GBP 4323
Total cost per participant	GBP 1312	GBP 1312	GBP 1312
SROI ratio (rounded to nearest 10 pence)	GBP 4.70:GBP 1	GBP 3.50:GBP 1	GBP 3.30:GBP 1

**Table 11:** Organisational overheads for the MW programme and estimated 12-month costs.

Organisational Overhead	Cost Description	Estimated Cost (GBP)
Trustee Board (Governance)	One face-to-face meeting per annum. A total annual volunteering opportunity cost for Trustee time of GBP 1076.96 is based on an estimated opportunity cost of GBP 195.81 per hour and 5.5 h of meetings per year divided between three Fathom programmes.	GBP 50 (GBP 150/3 programme) GBP 359 ((GBP 195.81 × 5.5 h)/3 programmes)
Programme Development Board (PDB)	Two face-to-face meetings per annum. A total annual volunteering opportunity cost for PDB time of GBP 1449 is based on an estimated opportunity cost of GBP 207 per hour and seven hours of meetings per year divided between three Fathom programmes.	GBP 80 (GBP 240/3 programmes) GBP 483 ((GBP 207 × 7)/3 programmes)
Programme management	Charity Director estimated hourly rate GBP 23.36 × 324 h management time per annum.	GBP 7568.20 (GBP 23.36 × 324 h)
Programme administration	Admin Assistant estimated hourly rate GBP 9.93 × 324 h administration time per annum.	GBP 3217.32
Fundraising (to ensure long-term sustainability)	Charity Director estimated hourly rate GBP 23.36 × 70 h fundraising time per annum.	GBP 1635.20
Craft maker Continued professional development	Relevant training courses @ GBP 625 per annum and development day @ GBP 400 divided between three programmes.	GBP 208.33 (GBP 625/3) GBP 133.33 (GBP 400/3)
Website for communication and marketing	Website hosting @ GBP 6 per month × 12 months, divided between three programmes.	GBP 24 (GBP 72/3)
**TOTAL (rounded to nearest GBP):**		**GBP 13,758**

**Table 12:** Forecast total costs to deliver 12 Making Well programmes over a 12-month period, including direct programme costs and 20% organisational overheads.

Cost Category	Cost Description	Cost Source	Estimated Cost per Day (GBP)	Nine-Week Programme Costs (GBP)	12-Month Costs (GBP) (12 MW Programmes)	Cost per Participant (GBP) (*n* = 144)
**Overhead costs**						
Projected 20% organisational overheads	See organisation cost methodology outlined above.	See above.			GBP 21,423.68	GBP 148.78
**Direct costs**						
Site hire	A mid-point cost (GBP 75 per day) of hire/rent of programme locations ranging from local, rural village halls (average GBP 50 per day) to Llanfellte Farm (GBP 100 per day) is applied.	Fathom Trust	GBP 75 (Estimated mid-point cost)	GBP 675	GBP 8100	GBP 56.25
Outdoor heating	It is estimated that 4 fire bowls (GBP 150 each) and firewood for six programmes running October to March (GBP 20 per programme) will be required.	Fathom Trust			GBP 720 ((GBP 150 × 4) + (GBP 20 × 12))	GBP 5
Catering	12 participants + 8 staff and volunteers at GBP 8 per person.	Fathom Trust	GBP 160 (GBP 8 × 20 people)	GBP 1440	GBP 17,280	GBP 120
Craft and horticulture sessions (Staff, equipment, materials)	Craft maker time, tools and materials cost GBP 250 per craft per day. 3 craft makers deliver sessions on the taster day and 2 craft makers deliver craft sessions each day of the eight-week programme, giving 19 craft sessions in total per programme.	Fathom Trust		GBP 4750 (GBP 250 × 19 craft sessions)	GBP 57,000	GBP 395.83
Volunteer time	10 volunteer hrs are required to support each day of the programme. Volunteering opportunity costs are estimated using the National Living Wage of GBP 9.50 per hour.	gov.uk/national-minimum-wage-rates [34]	GBP 95 (GBP 9.50 × 10 h)	GBP 855	GBP 10,260	GBP 71.25
**TOTAL COSTS (Overhead and Direct costs, rounded to the nearest GBP):**					**GBP 114,784**	**GBP 797**

**Table 13:** Forecast SROI ratios for 12 Making Well programme delivered over 12 months, based on learnings from six-month pilot and Fathom Trust commitments going forwards.

	Well-Being Valuation Approach		
	Social Value Calculator Applied to Improved Overall Well-being	Social Value Calculator Applied to Increased Feeling of Belonging and Increased Confidence	Mental Health Social Value Calculator Applied to SWEMWBS Scores
Outcomes	Improved overall well-being	Higher confidence and Increased sense of belonging (social connection)	Improved mental health
Well-being-related Social Value per participant	GBP 6107	GBP 4538	GBP 4290
NHS cost saving per participant	GBP 33	GBP 33	GBP 33
Total Social Value per participant	GBP 6140	GBP 4571	GBP 4323
Total cost per participant	**GBP 797**	**GBP 797**	**GBP 797**
SROI ratio (rounded to nearest 10 pence)	**GBP 7.70:GBP 1**	**GBP 5.70:GBP 1**	**GBP 5.40:GBP 1**

## Data Availability

In the interests of data protection, the final dataset will only be available to the study investigators and the project team.

## Supplementary Materials

The following supporting information can be downloaded at: https://www.mdpi.com/article/10.3390/ijerph22081184/s1, Table S1: Estimated annual salaries, hourly rates and total volunteering opportunity cost per hour for The Fathom Trust board of Trustees; Table S2: Estimated annual salaries, hourly rates and total volunteering opportunity cost per hour for The Fathom Trust Programme Development Board.
