# Supplementary material for: Health Economics-Informed Social Return on Investment (SROI) Analysis of a Nature-Based Social Prescribing Craft and Horticulture Programme for Mental Health and Well-Being
Source: Int J Environ Res Public Health. 2025 Jul 29;22(8):1184. doi: 10.3390/ijerph22081184 (PMC12386064; doi:10.3390/ijerph22081184)
Supplement: Supplementary file 1 [file ijerph-22-01184-s001.zip › ijerph-3635904-supplementary.pdf]

Supplementary material

Table S1. Estimated annual salaries, hourly rates and total volunteering opportunity cost per hour for The Fathom Trust board of Trustees.

**Trustee Board member job title**	**Estimated average or mid-point annual salary**	**Estimated hourly rate***	**Salary source**
Chairman	£71,216	£37.53	https://www.payscale.com/research/UK/Job=Chairman/Salary
Chief Medical Scientist	£93,598	£49.33	https://www.payscale.com/research/UK/Job=Chief_Scientific_Officer_(CSO)/Salary
Economist	£38,329	£20.20	https://www.payscale.com/research/UK/Job=Economist/Salary
Director, NHS	£100,905	£53.18	Mid-point NHS Band 9 (£93,735 to £108,075), https://www.nhsemployers.org/articles/pay-scale-material-202223
Psychiatrist	£67,502	£35.57	https://www.payscale.com/research/UK/Job=Psychiatrist/Salary
**TOTAL ESTIMATED OPPORTUNITY COST PER HOUR:**		**£195.81**	

*Estimated hourly rates are based on 235 working days per year and 7.5 working hours per day.

Table S2. Estimated annual salaries, hourly rates and total volunteering opportunity cost per hour for The Fathom Trust Programme Development Board.

**Programme Development Board Sector representative**	**Expected job role/ title**	**Estimated average or mid-point annual salary**	**Estimated hourly rate***	**Salary source**
Academia (Evaluation & Learning)	Professor	£75,167	£39.61	https://www.payscale.com/research/UK/Job=Professor%2C_Postsecondary_%2F_Higher_Education/Salary
Children & Young people	Youth Worker	£22,236	£11.72	https://www.payscale.com/research/UK/Job=Youth_Worker/Salary
Conservation	Ecologist	£23,366	£12.31	https://www.payscale.com/research/UK/Job=Ecologist/Salary

Supplementary material

Craftsmanship	Charity Director	£44,343	£23.37	https://www.payscale.com/research/UK/Job=Executive_Director%2C_Non-Profit_Organization/Salary
Culture (Arts Council)	Arts and Health Coordinator	£24,296	£12.80	https://www.payscale.com/research/UK/Job=Community_Outreach_Coordinator/Salary
Health (NHS)	Primary Care Cluster Manager	£70,769	£37.30	Mid-point NHS Band 8c (£65,664 to £75,874), https://www.nhsemployers.org/articles/pay-scale-material-202223
Safeguarding (Local Authority)	Director for adult social care	£99,546	£52.46	https://www.glassdoor.co.uk/Salary/Civil-Service-United-Kingdom-Director-Salaries-E214431_D_KO29,37.htm
Soul/ Spiritual growth (Church)	Senior Pastor	£33,088	£17.44	https://www.payscale.com/research/UK/Job=Senior_Pastor/Salary
**TOTAL ESTIMATED OPPORTUNITY COST PER HOUR:**			**£207**	

*Estimated hourly rates are based on 235 working days per year and 7.5 working hours per day.
